# Bromatological Profile of Fruits from *Sorbus aucuparia* and *Crataegus monogyna*: Polyphenol Bioaccessibility and Inhibitory Effect on Lipid Peroxidation in a Biological Model

**DOI:** 10.3390/antiox15030349

**Published:** 2026-03-11

**Authors:** Iulia Varzaru, Arabela Elena Untea, Petru Alexandru Vlaicu, Alexandra Gabriela Oancea, Raluca Paula Turcu

**Affiliations:** Feed and Food Quality Department, National Research and Development Institute for Animal Biology and Nutrition, 077015 Balotesti, Ilfov, Romania; arabela.untea@ibna.ro (A.E.U.); alexandra.oancea@ibna.ro (A.G.O.); raluca.turcu@ibna.ro (R.P.T.)

**Keywords:** berries, antioxidants, bioaccessibility, polyphenols, lipid peroxidation, antioxidant activity, nutraceuticals, phytochemicals, health-promoting properties

## Abstract

This study presents a comprehensive analysis of the bromatological profile of fruits from rowanberry (*Sorbus aucuparia* L.) and hawthorn (*Crataegus monogyna* Jacq.), as well as the polyphenol bioaccessibility under in vitro simulated gastrointestinal conditions, antioxidant activity and the inhibition of lipid peroxidation in a biological model (egg yolk). The fruits were demonstrated to be rich in bioactive compounds, containing comparable total vitamin E levels (~65 mg/kg), with α-tocopherol as the predominant isomer, and measurable amounts of xanthophylls, mainly lutein (20.19–21.69 μg/g), astaxanthin, and canthaxanthin. HPLC-DAD analysis identified 19 polyphenolic compounds, with catechin being the dominant compound in rowanberry fruits (4.36 mg/g), while epigallocatechin and catechin were the most abundant in hawthorn fruits. In vitro gastrointestinal digestion showed elevated intestinal bioaccessibility of hydroxybenzoic acids, with ellagic acid reaching ~96% in the intestinal phase of rowanberry fruits and ~109% in hawthorn fruits, indicating increased availability. In hawthorn fruits, flavanols exhibited greater stability and higher bioaccessibility, with catechin reaching 101% in the gastric phase, epicatechin remaining highly bioaccessible (98–97%), and epigallocatechin showing moderate bioaccessibility (24–50%). Both fruit extracts exhibited antioxidant activity, with hawthorn fruits showing significantly higher ABTS and DPPH scavenging capacities. Rowanberry and hawthorn fruits exhibited an inhibitory effect on lipid peroxidation in yolk homogenates, reducing malondialdehyde formation to 37.19 mg/kg and 20.58 mg/kg from 50.79 mg/kg, respectively, although their efficacy remained lower than that of synthetic antioxidants. The findings of this study indicate that rowanberry and hawthorn fruits are promising sources of bioactive compounds, exhibiting significant antioxidant activity in biological models and supporting the potential valorization of these underutilized fruits for functional food and nutraceutical applications.

## 1. Introduction

The increasing demand of consumers for natural and plant-based products with health-promoting properties has led to the search for natural alternatives to synthetic additives [1]. Wild berries are known as valuable fruits, rich in nutrients and bioactive compounds that can prevent numerous illnesses. The health-benefits of berries are mainly attributed to synergistic, complementary, or additive interactions between their high content of nutrients (such as vitamins, minerals, folate, and dietary fiber) and the wide diversity of phytochemicals they contain, particularly phenolic compounds. Nevertheless, despite their high phytonutrient content, a wide range of woodland berries remain underused, due to the lack of knowledge on their nutritional value, underestimation of their potential use, or due to their disagreeable flavor, as sensed by consumers [2]. Research for integrating these underutilized valuable resources into nutritional strategies for consumption could generate substantial benefits and new opportunities in animal nutrition, agri-food, and nutraceutical and pharmaceutical industries.

Rowanberry (*Sorbus aucuparia*) and hawthorn (*Crataegus monogyna*), both belonging to the Rosaceae family, are underused species from wild flora. Rowanberry is a slender tree that can reach heights of 15–20 m. It is widespread in the Northern Hemisphere, occurring in cold regions and at high altitudes, and is also used for decorative purposes in gardens and parks. Rowanberries have a spherical shape and are reddish-orange in color. They contain 2–3 elongated stones and have small macules known as lenticels, which are red or brown when ripe [1,2]. Hawthorn is a semi-evergreen shrub or small tree with thorns, reaching heights of 5–15 m. It is widely distributed in the Northern Hemisphere, especially in mountainous and hilly regions. When ripe, the hawthorn fruit is less than 1 cm in diameter and contains a single small seed [3].

Their reddish fruits have been known for centuries for their nutritional and medicinal properties, being consumed as foods or as ingredients in phytotherapeutic preparations [4]. Hawthorn fruits have been traditionally used in cardiovascular therapy due to their antispasmodic, cardiotonic, hypotensive, and anti-atherosclerotic effects. Rowanberries were used as anti-inflammatory, antidiarrheal, diuretic, and vasodilator agents [5]. Their efficiency in gastrointestinal, pulmonary, renal, hepatic, and cardiovascular diseases is also documented [2].

The medicinal uses of rowanberry and hawthorn fruits are based on their phytochemical composition. Hawthorn has fruits rich in organic acids (malic, citric and oxalic acids), polyphenols (epicatechin, chlorogenic acid, and quercetin), mucoxanthin, triterpenoids, trace elements (Cu, Fe, Mg, Mn and Zn), and ascorbic acid [1,6]. Compared to other berries like jostaberry, lingonberry, or aronia, rowanberry stands out as the fruit with the highest total analyzed organic acid content, with malic acid being the main one [7]. Although it contains a significant content of phytochemicals, such as phenolic acids (chlorogenic, neochlorogenic and caffeic acids), vitamins (vitamin C, vitamin E), carotenoids (β-carotene, zeaxanthin, β-cryptoxanthin, all-trans-β-carotene), and minerals (K, P, Ca, Mg, Fe, Cu) [8], rowanberry remains underused due to the fact that it has an astringent flavor, which is less pleasant for consumers, being caused by the content of parasorbic acid. However, thermal processing or freezing converts parasorbic acid into harmless sorbic acid, which is suitable as a sweetener for diabetics [5].

The effects of polyphenols observed in vivo are largely dependent on their bioaccessibility and bioavailability after ingestion. Bioavailability is defined as the fraction of a compound that is digested, absorbed, and metabolized, and it depends on the amount of the absorbed compound. Bioaccessibility refers to the amount of an ingested nutrient that is available for absorption in the human gastrointestinal tract [9]. Several studies have shown that polyphenol bioaccessibility is associated with their molecular weight [10], and is influenced by the interaction of the polyphenols with other food components, like fibers that may reduce or improve their bioavailability in the gut [11]. Thus, it is important to consider that the antioxidant properties of the polyphenols depend not only on their structure and concentrations in foods but also on their bioaccessibility, as poor bioaccessibility results in reduced health effects.

Hawthorn fruits have been previously investigated, but limited information on the digestive fate and bioaccessibility of individual polyphenols is provided. In contrast, data on rowanberries remain scarce, especially regarding in vitro digestion and biological antioxidant efficacy. The parallel investigation of these two species therefore allows not only a direct comparison of the raw compositional profiles of a relatively well-studied and a less-studied fruit, but also an assessment of how structurally distinct yet taxonomically related fruits behave under identical digestive conditions. Therefore, it was hypothesized that differences in fruit structure and matrix composition between *C. monogyna* and *S. aucuparia* may influence the digestive fate and bioaccessibility of individual polyphenols under in vitro gastrointestinal conditions.

In the post-pandemic context, characterized by increased consumer awareness of health, nutrition, and sustainable food sources, the aim of this study was to provide a comparative bromatological profile of rowanberry and hawthorn fruits, as well as to evaluate the polyphenol bioaccessibility under in vitro simulated gastrointestinal conditions, antioxidant activity and the inhibition of lipid peroxidation in a biological model (egg yolk). This research contributes to a better understanding of their nutritional value and potential health benefits and seeks to promote the valorization of these underused fruits in the context of sustainable and health-oriented food systems.

## 2. Materials and Methods

### 2.1. Plant Material

The experimental material consisted of rowanberry (*S. aucuparia*) and hawthorn (*C. monogyna*) fruits, which were supplied by a local commercial producer (Din Bărăgan, Mihail Kogălniceanu, Romania) as organic cultivars from Ialomița County (44°35′53.88″ N, 27°12′22.68″ E), Romania. All analyzed fruits originated from a single harvest season, were collected at comparable ripeness stages, and were supplied by the same local producer, in order to reduce the variability related to harvest time and maturity. The fruits were delivered in dried form and packaged in zip-lock bags, and were ground whole, without seed removal, using a Grindomix GM 200 mill (Retsch, Haan, Germany) with a 0.5 mm sieve to obtain a fine powder. Subsequently, average samples were formed and stored in a dry and dark environment at room temperature to preserve their quality until analysis.

### 2.2. Analytical Standards and Reagents

The standards for polyphenols, xantophylls and isomers of vitamin E were supplied by Sigma-Aldrich (Darmstadt, Germany). FAME (Fatty Acid Methyl Esters) as a standard mixture was purchased from Merck (Darmstadt, Germany).

1,1-Diphenyl-2-picrylhydrazyl (DPPH), 2,2′-azino-bis-(3-ethylbenzthiazoline-6-sulphonic acid) radical (ABTS+), butylated hydroxytoluene (BHT), disodium ethylenediaminetetracetate (EDTA), ferric chloride, and ascorbic acid (AA) were purchased from Sigma-Aldrich (Darmstadt, Germany). Ethanol, methanol, acetone and acetic acid were obtained from Sigma-Aldrich (Darmstadt, Germany). All other reagents used to prepare the simulated saliva fluid, simulated gastric fluid, and simulated intestinal fluid were of analytical grade and provided by Sigma-Aldrich (Darmstadt, Germany).

### 2.3. Chemical Analysis

#### 2.3.1. Proximate Composition

The basic chemical composition of rowanberry and hawthorn fruits was determined through standardized analytical procedures. Total protein content was quantified by the Kjeldahl nitrogen method in accordance with ISO 5983-2:2009 [12], using a Kjeltec Auto 1030 system. Lipid content was measured by continuous ether extraction following SR ISO 6492:2001 [13] with Soxtec 2055 equipment (FOSS Analytical AB, Höganäs, Sweden). Crude fiber was assessed using the intermediate filtration technique as specified in SR EN ISO 6865:2002 [14], employing a Fibertec 2010 system. Dry matter and total ash contents were established gravimetrically according to ISO 6496:2001 [15] and ISO 2171:2010 [16], respectively, and were carried out in a Nabertherm calcination furnace (Nabertherm GmbH, Lilienthal, Germany).

The total carbohydrate content of fruits was calculated using the following equation [17]:

(1) Total carbohydrate (%) = 100 − [moisture (%) + ash (%) + fiber (%) + protein (%) + fat (%)]

#### 2.3.2. Minerals Analysis

The concentrations of zinc, iron, copper, and manganese were quantified by flame atomic absorption spectrometry using a SOLAAR M6 Dual Zeeman Comfort instrument (Thermo Electron, Cambridge, UK) following microwave-assisted sample digestion. All analytical procedures were conducted in accordance with a previously established protocol [18]. Phosphorus content was determined separately by a spectrophotometric assay based on colorimetric detection, with absorbance measurements recorded using a UV–Vis spectrometer (Thermo Electron, Cambridge, UK).

#### 2.3.3. Fatty Acids Analysis

The assessment of the fatty acid profile was performed using a gas chromatographic method [19]. The fatty acids were converted to fatty acid FA methyl ester. The samples were analyzed using a Perkin Elmer Clarus 500 gas chromatograph (Waltham, MA, USA). The chromatographic column was a capillary separation column with a high polar stationary phase TRACE TRFame, (Thermo Electron, Waltham, MA, USA), with dimensions of 60 m × 0.25 mm × 0.25 mm. For detection, a flame ionization detector (FID, Thermo Electron, Cambridge, UK) was used, and identification and quantification were achieved by referencing analytical standards. The results were expressed for each FA as % of total Fatty Acid Methyl Esters (FAME).

#### 2.3.4. Liposoluble Antioxidants Analysis

For the preparation of extracts for the analysis of liposoluble compounds, saponification was performed using an ethanolic potassium hydroxide solution for 30 min at 80 °C in a water bath. After saponification, the samples were cooled at room temperature. Subsequently, repeated extractions were carried out with petroleum ether. The combined extracts were passed through a filter with anhydrous sodium sulfate to remove residual moisture and evaporated under vacuum to dryness. The resulting residue was reconstituted in 10 mL of ethanol. Prior to HPLC analysis, each of the analyzed samples underwent filtration using a 0.45 μm membrane filter acquired from Teknokroma (Barcelona, Spain).

Xanthophyll analysis, including lutein, astaxanthin, and canthaxanthin, was carried out following a previously reported protocol [20]. Separation and detection were achieved using a Surveyor Plus high-performance liquid chromatography system (Thermo-Electron Corporation, Waltham, MA, USA) equipped with a photodiode array UV detector and fitted with a reversed-phase C18 column (250 × 4.60 mm, 5 μm; Nucleodur, Macherey-Nagel, Düren, Germany). The chromatographic column was maintained at 25 °C during the analysis. The mobile phases consisted of 10% water, 15% methanol, and 75% acetone, in isocratic conditions, with a flow rate of 0.5 mL/min. The injection volume was 25 μL and the chromatograms were registered at 450 nm. The individual carotenoids were identified and quantified using individual analytical standards. The results were expressed as μg/g.

The analysis of vitamin E isomers was conducted from the same extracts prepared for the carotenoids analysis. Separation and quantification were performed using a Vanquish Core HPLC system coupled to a diode array detector (Thermo-Electron Corporation, Waltham, MA, USA). An Accucore C18 column (150 mm × 4.6 mm, 4 μm particle size) (Thermo-Electron Corporation, Waltham, MA, USA) was employed for chromatographic separation. The mobile phases consisted of methanol (96%) and water (4%), with a flow rate of 0.5 mL/min and isocratic conditions. The injection volume was 40 μL. The detection was performed at 292 nm. The results were expressed as μg/g.

#### 2.3.5. Polyphenol Analysis

HPLC-DAD analysis was employed to investigate the phenolic composition of fruit extracts, which were prepared as previously described [10]. The detailed procedure for the preparation of the extracts for polyphenol analysis is provided in the Supplementary Materials. For each digestive phase (oral, gastric, and intestinal phase), a 1 mL aliquot part was passed through an SPE cartridge and prepared in the same conditions as for solid samples. The HPLC system consisted of a Vanquish Core HPLC system coupled to a diode array detector (Thermo-Electron Corporation, Waltham, MA, USA). Separation of the extract constituents was carried out on a BDS HyperSil C18 reversed-phase column (250 × 4 mm, 5 μm; Thermo Fisher Scientific, Bremen, Germany). The chromatographic system employed a mobile phase consisting of aqueous acetic acid (1%, *v*/*v*; solvent A), methanol (solvent B), and acetonitrile (solvent C), delivered at a constant flow rate of 0.5 mL/min. Gradient elution was applied according to the following program: 0–15 min, 5% B and 5% C; 15–20 min, 4% B and 15% C; 20–25 min, 3% B and 25% C; 25–40 min, 2% B and 38% C; followed by re-equilibration from 40 to 50 min at 5% B and 5% C. Samples (40 μL) were injected, and the column temperature was controlled at 25 °C. Detection was performed by monitoring UV absorbance at 254, 270, 280, 310, and 320 nm. Quantitative analysis of the identified analytes was conducted using external calibration curves.

#### 2.3.6. Antioxidant Activity Analysis

The extracts of selected fruits were prepared with 80% methanol, following the method described in the Supplementary Materials. Four different spectrophotometric methods were used for the determination of DPPH, ABTS, antioxidant capacity, and iron chelating ability. The DPPH radical scavenging activity of the fruit extracts was assessed following a previously described spectrophotometric method [21] and Varioskan Lux microplate reader (Thermo Fisher Scientific, Waltham, MA, USA). The calibration curve was plotted using 6-hydroxy-2,5,7,8 tetramethylchroman-2-carboxylic acid (Trolox) and the results were expressed as mmol Trolox equivalents/kg of the sample.

The phosphomolybdenum method was also used to evaluate the total antioxidant activity (TAC) of the fruit extracts. The absorbance of the samples was measured at 695 nm, and the results were expressed as mM ascorbic acid equivalents (mM AAE). The scavenging activity against the ABTS radical cation (2,2′-azino-bis(3-ethylbenzothiazoline)-6-sulphonic acid) was determined using the method described by Untea et al. [22]. ABTS•+ was produced by reacting 7 mM ABTS and 2.4 mM potassium persulphate in the same ratio. The mixture was kept in the dark for 12 h at room temperature, allowing it to react. ABTS solution was further diluted with ethanol to an absorbance of 0.7 at 734 nm. Sample solution was added to ABTS solution, and the absorbance was measured at 734 nm (Jasco V-530, Japan Servo Co., Ltd., Tokyo, Japan) using ethanol as blank. The ABTS radical scavenging activity was expressed as millimoles of Trolox equivalents per kg.

The ability of rowanberry and hawthorn fruit extracts to chelate Fe^2+^ ions was assessed using a modified ferrozine-based assay. Briefly, 1 mL of the methanolic extract (1:10, *w*/*v*) was placed in a 10 mL volumetric flask and combined with 1.6 mL of deionized water. A freshly prepared FeCl_2_ solution (2 mM; 0.06 mL) was then introduced, and the reaction mixture was maintained at room temperature for 3 min. The chelation reaction was subsequently developed by adding 0.12 mL of ferrozine solution (5 mM). Following thorough agitation, the mixture was incubated for 10 min in ambient conditions. Absorbance corresponding to the formed complex was recorded at 562 nm using a UV–Vis spectrophotometer, with the blank treated identically but without extract. The results were expressed as milligrams of EDTA equivalents per gram of sample (mg EDTA eq/g).

### 2.4. Simulated In Vitro Gastrointestinal Digestion of the Rowanberry and Hawthorn Fruits

In vitro digestion of rowanberry and hawthorn fruits was conducted according to the method published earlier [10], which consisted of oral, gastric and intestinal phases. The detailed procedure for the preparation of the digestion phases is provided in the Supplementary Materials. For the oral phase, 5 g of powdered sample were dispersed in simulated salivary fluid (3.5 mL), followed by the addition of pre-heated α-amylase solution (0.5 mL, 75 U/mL in SSF, 37 °C). Calcium chloride solution (25 μL, 0.3 M) and distilled water (975 μL) were then incorporated to complete the reaction mixture. After thorough homogenization, the mixture was incubated at 37 °C for 2 min.

The resulting oral bolus was subsequently subjected to gastric digestion by mixing with pre-heated (37 °C) simulated gastric fluid (7.5 mL) and pepsin solution (1.6 mL, 2000 U/mL in SGF). A defined amount of CaCl_2_ solution (5 μL, 0.3 M) was incorporated, and the pH was adjusted to 3 using 6 M HCl. Distilled water was added to achieve a total volume of 10 mL. The gastric mixture was incubated at 37 °C for 2 h.

For the intestinal phase, the gastric chyme was combined with simulated intestinal fluid (11 mL), pancreatin solution (5 mL, 800 U/mL in SIF; final activity 100 U/mL), bile salt solution (2.5 mL, 160 mM; final concentration 10 mM), and CaCl_2_ (40 μL, 0.3 M). The pH was raised to 7 using 1 M NaOH, and distilled water was added to obtain a final 1:1 (*v*/*v*) ratio between intestinal fluids and gastric chyme. The intestinal digestion was carried out at 37 °C for 2 h. At the end of each digestion stage, samples were centrifuged at 4500 rpm for 15 min at 4 °C using a refrigerated centrifuge (2-16KL, Sigma Laborzentrifugen GmbH, Osterode am Harz, Germany). The supernatants were collected and subjected to HPLC analysis in order to determine the bioaccessibility of individual polyphenolic compounds.

The bioaccessibility index (BI), referred to as the fraction of an ingested compound that becomes potentially available for absorption during the digestion process, was estimated using the following Formula [23]:

(2) BI (%) = (Phenolic content released during digestion)/(Phenolic content before digestion) × 100

### 2.5. Lipid Peroxidation Inhibition Assay

To study the potential of the fruit extract to inhibit lipid peroxidation in a biological model based on an egg yolk homogenate, a method for iron-induced lipid oxidation was used [24]. Peroxidation was initiated by adding FeCl_2_ (100 μM) and ascorbic acid (500 μM) to the egg yolk homogenate, with or without plant methanolic extracts (at a concentration of 1000 mg L^−1^)/synthetic antioxidant (EDTA/BHT/vitamin E). The mixture was incubated at 37 °C for 60 min.

Lipid peroxidation in egg yolk homogenates was quantified through the determination of thiobarbituric acid reactive substances (TBARS). Absorbance readings were recorded spectrophotometrically using a UV–Vis instrument calibrated with a standard curve prepared from 1,1,3,3-tetramethoxypropane. The extent of lipid oxidation was calculated as malondialdehyde equivalents and reported as micrograms of MDA per kilogram of sample (μg MDA/kg).

The percentage of lipid peroxidation inhibition was calculated using the following formula:

(3) % Inhibition = (AC − AS) × 100/AC

where AS was the sample absorbance and AC was the control of oxidation absorbance.

The relative inhibition of lipid oxidation was assessed as an efficacy factor (EF) and calculated by dividing the TBARS value of the peroxidized homogenate by that of the peroxidized homogenate with synthetic antioxidants or fruit extracts [25]. Higher values of efficiency factor indicate an increased effectiveness of the tested antioxidants in decreasing lipid oxidation in the egg yolk homogenate.

### 2.6. Statistical Analysis

For each fruit type, three independent samples were formed, and each sample was analyzed in triplicate. The data obtained were analyzed using one-way ANOVA, followed by Tukey’s test (*p* = 0.05) using XLSTAT software (v.19.01, Addinsoft, Paris, France). Prism GraphPad software v. 9.1.2 (San Diego, CA, USA) was used to compare the data regarding the intestinal bioaccessibility of the polyphenols, antioxidant capacity and inhibitory effect on lipid peroxidation.

## 3. Results

### 3.1. Proximate Composition and Mineral Content of Rowanberry and Hawthorn Fruits

The results regarding the proximate composition of the fruits of rowanberry and hawthorn are presented in Table 1. It was shown that hawthorn fruits had a significantly higher (*p* < 0.05) fiber content in their dry matter, while rowanberry fruits registered a significantly higher content (*p* < 0.05) of protein and fat compared to hawthorn fruits. Regarding ash content, a minor difference was observed between the two variants, but not significant. Additionally, the carbohydrate content was found to be significantly higher in rowanberry fruits.

The composition of major and trace mineral elements of the fruits of rowanberry and hawthorn is shown in Table 1. The highest (<0.0001) content of copper was found in hawthorn fruits, which also contained an elevated concentration of (<0.0001) iron and zinc. Rowanberry fruits registered the highest (<0.0001) concentration of manganese.

### 3.2. Fatty Acid Profile of Rowanberry and Hawthorn Fruits

The fatty acid composition of the two types of fruits is presented in Table 2. Both fruits showed a fatty acid profile dominated by polyunsaturated fatty acids (PUFAs), followed by monounsaturated (MUFAs) and saturated fatty acids (SFAs), although significant quantitative differences were observed between the analyzed fruits. The major components were linoleic acid (C18:2n-6), oleic acid (C18:1n-9) and palmitic acid (C16:0). Smaller amounts of stearic (C18:0) and tricosanoic acid (C23:0) were also identified in the samples of rowanberry and hawthorn fruits.

Many individual saturated fatty acids (SFAs) had no significant variation between the two fruits. However, palmitic acid (C16:0), the predominant SFA in both species, exhibited a significantly higher concentration in hawthorn fruits, contributing to the overall higher SFA content (18.66 g/100 g total Fas) compared with rowanberry fruits (15.57 g/100 g).

The fraction of monounsaturated fatty acid (MUFA) was relatively similar in the two species, with no significant differences in the major MUFA—oleic acid (C18:1n-9). Important differences were registered in the distribution of omega fatty acids. Hawthorn fruits showed significantly higher concentrations of total n-3 fatty acids (Σ n-3), particularly α-linolenic acid (C18:3n-3), octadecatetraenoic acid (C18:4n-3), and eicosapentaenoic acid (C20:5n-3), leading to a markedly lower n-6/n-3 ratio compared to rowanberry (19.31 vs. 44.39; *p* < 0.0001). In contrast, rowanberry fruits were characterized by a higher total n-6 fatty acid content, mainly due to increased linoleic and γ-linolenic acid levels.

The PUFA/SFA ratio was significantly higher in rowanberry fruits (3.69) compared with hawthorn fruits (2.91). The n-6/n-3 ratio was more balanced in hawthorn fruits. From a nutritional perspective, this ratio is considered a meaningful indicator of dietary lipid quality, with lower values generally associated with more favorable health outcomes. The analytical results showed that, although hawthorn fruits contained higher levels of SFAs, they also exhibited a higher n-3 fatty acid content and a reduced n-6/n-3 ratio compared with rowanberry fruits.

### 3.3. Liposoluble Antioxidants of Rowanberry and Hawthorn Fruits

**Figure 1:**
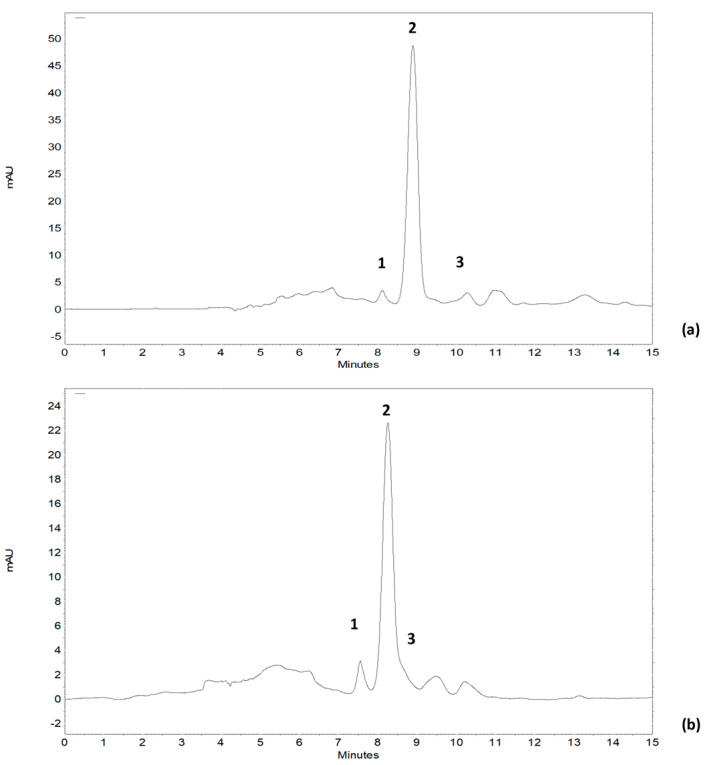
Identification of individual carotenoids found in rowanberry fruits (**a**) and hawthorn fruits (**b**) 1—astaxanthin, 2—lutein, 3—canthaxanthin.

Individual xanthophylls of the samples were identified by HPLC-DAD and two of the most representative chromatograms are shown in Figure 1.

The results of the quantitative analysis of lutein, astaxanthin, and canthaxanthin are presented in Table 3, together with the tocopherol profile of rowanberry and hawthorn fruits.

The rowanberry fruits show a total vitamin E content of 65.39 mg/kg, very close to that measured in hawthorn fruits, which is 65.70 mg/kg. In both fruits, the predominant isomer is α-tocopherol, with 34.07 mg/kg in rowanberry and 47.70 mg/kg in hawthorn. α-Tocopherol is the most biologically active form of vitamin E. Both fruits also contain significant amounts of δ-tocopherol and γ-tocopherol, which contribute to their antioxidant activity. Hawthorn fruits exhibited statistically significantly higher (*p* < 0.05) concentration of α-tocopherol, whereas rowanberries showed a significantly higher (*p* < 0.05) level of γ-tocopherol. These values highlight that both fruits are good sources of vitamin E with important antioxidant potential.

Regarding carotenoids, both rowanberry and hawthorn fruits contain similar amounts of lutein (approximately 20–22 mg/kg) and astaxanthin (about 0.87–0.91 mg/kg). Additionally, the combined lutein and zeaxanthin content is higher in hawthorn fruits (141.52 mg/kg) compared to rowanberry (130.60 mg/kg). These carotenoids are known for their antioxidant properties and important roles in eye health protection, further enhancing the nutritional value of these fruits.

### 3.4. Polyphenol Profile and In Vitro Bioaccessibility Assessment

**Figure 2:**
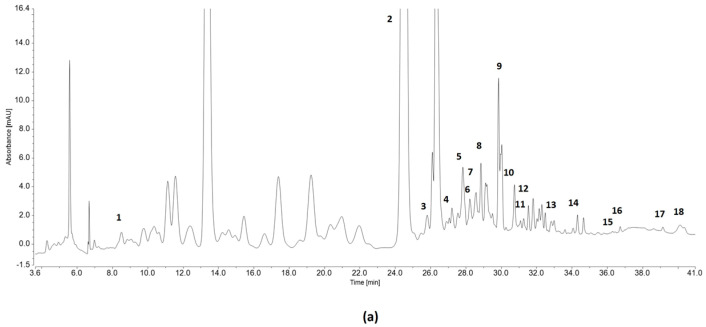

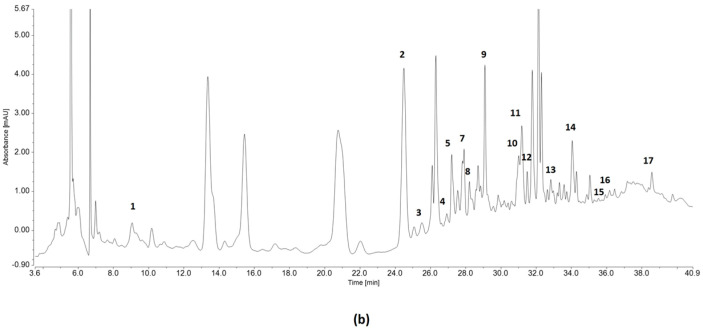
Chromatograms of phenolic compounds extracted from rowanberry fruits (**a**) and hawthorn fruits (**b**) before digestion. Peaks identification: 1—gallic acid, 2—epigallocatechin, 3—catechin, 4—chlorogenic acid, 5—vanillic acid, 6—caffeic acid, 7—syringic acid, 8—epicatechin, 9—3-hydroxybenzoic acid, 10—rutin, 11—coumaric acid, 12—ellagic acid, 13—p-methoxycinnamic acid, 14—ferulic acid, 15—protocatechuic acid, 16—resveratrol, 17—quercetin, 18—cinnamic acid.

The HPLC-DAD analysis of the rowanberry and hawthorn fruits allowed the identification of 19 polyphenolic compounds, namely gallic acid, epigallocatechin, catechin, chlorogenic acid, vanillic acid, caffeic acid, syringic acid, epicatechin, 3-hydroxybenzoic acid, rutin, coumaric acid, ellagic acid, p-methoxycinnamic acid, ferulic acid, protocatechuic acid, resveratrol, quercetin, and cinnamic acid (Figure 2).

The phenolic composition of the rowanberry and hawthorn fruits before and after simulated digestion is presented in Table 4 and Table 5. The subclass of flavanols was the most prominent in both types of fruit. Catechin was the most abundant polyphenol in rowanberry fruits, whereas in hawthorn fruits the highest content was observed for epigallocatechin, with values close to those of catechin. Trans-cinnamic acid was detected only in rowanberry fruits. Other compounds, like the stilbene resveratrol, were present in small amounts in both fruits, with lower values in rowanberry (0.001 mg/g) compared to hawthorn (0.004 mg/g).

It was observed that both fruits showed substantial changes in phenolic composition during digestion. In rowanberry fruits, most hydroxycinnamic acids show a moderate intestinal bioaccessibility. On the other hand, hydroxybenzoic acids registered elevated intestinal bioaccessibility, with ellagic acid reaching ~96% in the gastric and intestinal phases, and vanillic acid reaching ~104% in the intestinal phase, indicating an increased availability. Hawthorn exhibited much higher bioaccessibility index, especially in later digestion stages: gallic acid registered 164% BI in the intestinal phase, syringic acid and ellagic acid also exceeded 100%, indicating strong release or conversion during digestion.

Among flavanols, catechin remains highly stable in rowanberry fruits, showing a progressive increase in the gastric and intestinal phases, whereas epigallocatechin undergoes a major decline in the intestinal phase. Quercetin was found only in the intestinal phase in rowanberry fruits, which may be due to the deglycosylation of its glycosylated precursors, such as rutin. In hawthorn fruits, flavanols exhibited a better stability and higher bioaccessibility: catechin reached 101% in the gastric phase, epicatechin was highly bioaccessible (98–97%), while epigallocatechin showed moderate BI (24–50%), which was still higher than in rowanberry.

Regarding flavonols, in rowanberry fruits rutin registered a decreased BI in intestinal phase to only ~18% compared to 81% in hawthorn fruits. Quercetin was barely detectable in rowanberry intestinal phase, while 35% BI was registered in hawthorn intestinal phase. Anthocyanins were considerably more bioaccessible in hawthorn fruits, with over 130% in gastric and intestinal phases—indicating strong release or transformation of the phenolic compounds. In the case of resveratrol, both fruits exhibited elevated BI in the intestinal phase, with hawthorn values of 96–97% and rowanberry values of 93%, which were very similar.

**Figure 3:**
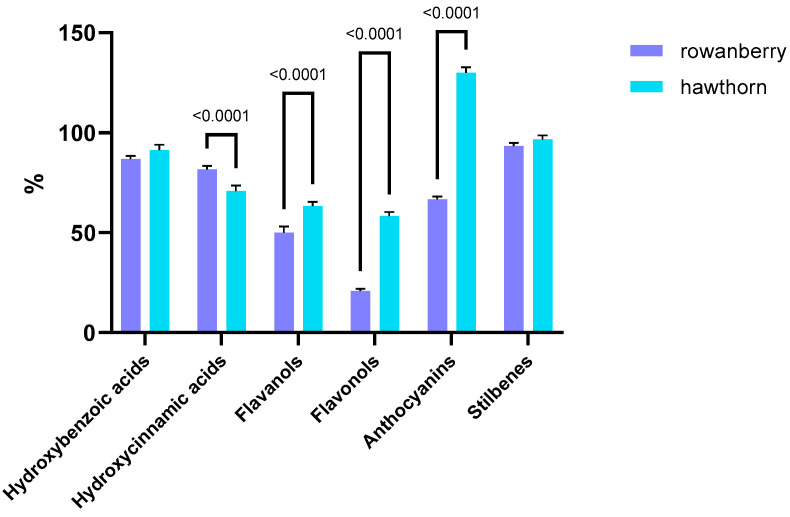
Intestinal bioaccessibility of polyphenol classes of rowanberry and hawthorn fruits.

The impact of gastrointestinal digestion on different classes of polyphenols is shown in Figure 3.

Class-dependent differences between the two fruit matrices were observed when the intestinal bioaccessibility profiles were plotted. Hawthorn consistently displayed higher intestinal bioaccessibility across most polyphenol classes. Hydroxybenzoic acids and stilbenes registered higher, although only numerically superior, intestinal bioaccessibility values, whereas significantly higher (*p* < 0.0001) bioaccessibility was observed for flavanols, flavonols and anthocyanins compared to rowanberry fruits. This may suggest a more favorable interaction between its fruit matrix and the digestive processes. Rowanberry fruits exhibited a significantly (*p* < 0.0001) increased value of the intestinal bioaccessibility of hydroxycinnamic acids, while the rest of the classes showed a comparatively lower intestinal bioaccessibility, suggesting a reduced stability or release of its polyphenolic constituents during digestion.

Overall, it was highlighted that hawthorn fruits had a generally more efficient release of polyphenols into the intestinal phase. Therefore, from a nutritional and functional perspective, hawthorn fruits may provide a higher effective availability of phenolic compounds after digestion compared to rowanberry fruits.

### 3.5. Antioxidant Capacity of Rowanberry and Hawthorn Fruits

**Figure 4:**
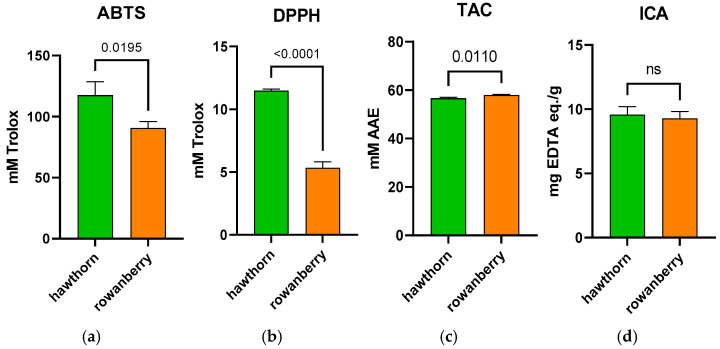
Antioxidant capacity of rowanberry and hawthorn fruits assessed by four different methods. (**a**) ABTS, (**b**) DPPH, (**c**) TAC—total antioxidant capacity, and (**d**) ICA—iron chelating ability. “ns” means “non-significant”.

The antioxidant capacity of rowanberry and hawthorn fruits was determined by four complementary assays (Figure 4), namely ABTS, DPPH, total antioxidant capacity (TAC), and iron chelating ability (ICA). The use of multiple analytical methods offers a comprehensive evaluation of antioxidant potential. Different assays target different antioxidant mechanisms, providing a holistic assessment of antioxidant capacity.

Hawthorn fruits exhibited significantly higher (*p* < 0.05) values for ABTS and DPPH, whereas rowanberry showed a significantly higher total antioxidant capacity (TAC; *p* = 0.0110). The evaluation of iron chelating ability revealed no significant differences between the two fruit types. In conclusion, both fruit types showed measurable antioxidant capacity across all assays, with differences observed depending on the method applied, which were strongly related to the composition and relative abundance of bioactive compounds responsible for radical scavenging, reducing power, and metal chelation.

### 3.6. Lipid Peroxidation Inhibition in a Biological Model

The extract’s capacity in preserving the foods from oxidation was assessed using egg yolk as a model. Table 6 presents the results regarding the antioxidant effect of the tested extracts in decreasing the MDA formation, a marker of lipid peroxidation.

As expected, the peroxidized homogenate (PH) registered a markedly elevated MDA level (50.79 mg/kg), confirming the successful induction of lipid peroxidation. The addition of synthetic antioxidants, such as EDTA, BHT, and vitamin E to the peroxidized homogenate resulted in significant (*p* < 0.05) reductions in MDA formation, with values ranging between 9.12 and 12.12 mg/kg, demonstrating the strong antioxidant efficacy of these widely used synthetic antioxidants. No significant differences were observed in the malondialdehyde values of the peroxidized homogenates supplemented with synthetic antioxidants.

Among the natural extracts evaluated, hawthorn (PHH) and rowanberry (PHR) exhibited a significant (*p* < 0.05) decreased concentration of MDA compared to peroxidized homogenate (PH). Hawthorn (PHH) showed a moderate inhibitory effect on lipid peroxidation (20.58 mg/kg), whereas rowanberry (PHR) exhibited weaker antioxidant protection, with an MDA level of 37.19 mg/kg, when compared to the selected synthetic antioxidants.

**Figure 5:**
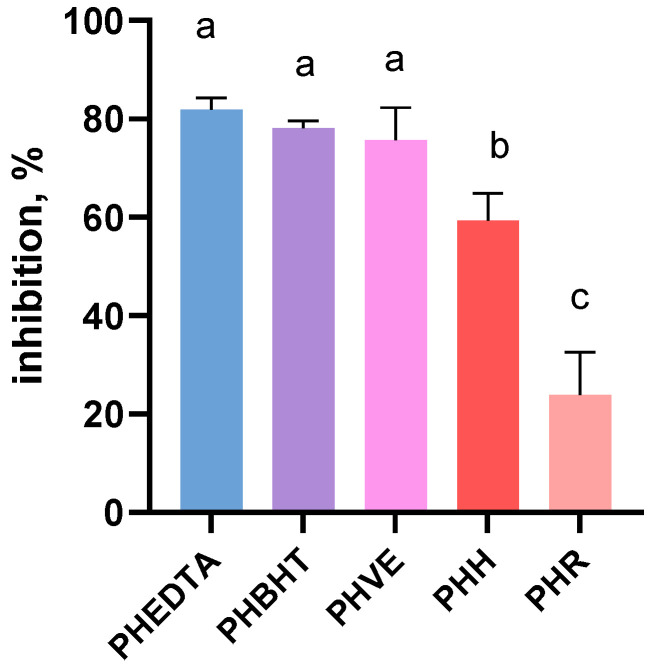
Inhibitory effect of rowanberry and hawthorn fruit extracts and synthetic antioxidants on lipid oxidation in a selected biological model (egg yolk). Different letters (a,b,c) show significant differences at *p* < 0.05 according to ANOVA test.

The inhibitory effect of rowanberry and hawthorn fruit extracts together with that of the selected synthetic antioxidants on induced lipid peroxidation in an egg yolk model is depicted in Figure 5. The figure compares the inhibition percents of lipid oxidation in egg yolk, highlighting the relative efficacy of each antioxidant in mitigating lipid oxidation.

It was observed that there was a clear hierarchy of antioxidant efficacy, with synthetic antioxidants outperforming the fruit extracts and hawthorn fruits displaying greater inhibitory potential than rowanberry fruits. Overall, the figure effectively highlights the differential antioxidant responses and is supported by the quantitative findings regarding the polyphenol bioaccessibility reported in this study.

**Figure 6:**
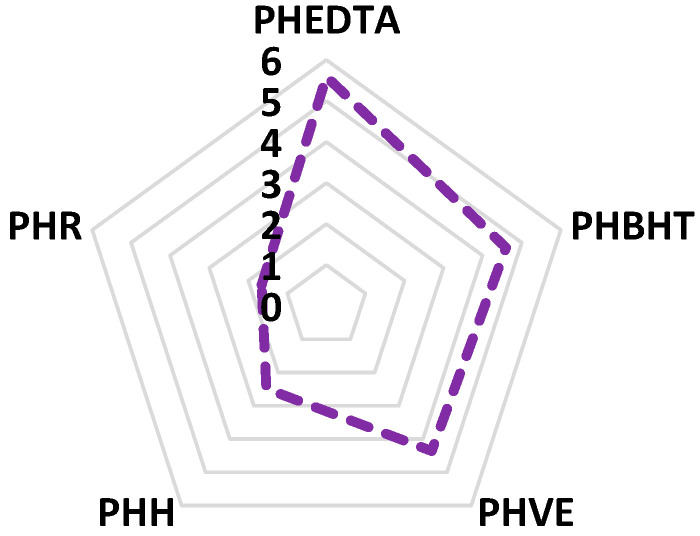
Relative prevention of lipid oxidation expressed as the efficiency factor (EF).

Figure 6 presents the efficiency factor (EF) of the studied antioxidants and extracts in relation to TBARS measurement of peroxidized homogenates. Higher EF values indicate a more pronounced reduction in TBARS formation, and thus a stronger protective effect against oxidative processes. As shown, peroxidized homogenates that contained EDTA, BHT and vitamin E displayed the highest efficiency, confirming their well-established potency in inhibiting lipid peroxidation. Regarding the fruit extracts, hawthorn demonstrates a moderate retarding effect on oxidative processes, whereas rowanberry exhibits a comparatively lower efficiency. Overall, the efficiency factor evaluation provides a clear comparative assessment of antioxidant efficacy within the selected experimental model.

## 4. Discussion

### 4.1. Comparative Assessment of the Bromatological Profile

The present study aims to provide a comparative assessment of the bromatological profile of rowanberry (*S. aucuparia*) and hawthorn (*C. monogyna*) fruits, highlighting important differences and similarities in their nutritional composition. Significant differences were found in the compositional attributes of the two fruit types.

The proximate analysis of the fruits revealed that rowanberry fruits were characterized by a significantly higher crude protein and crude fat content, whereas hawthorn fruits showed a markedly higher crude fiber content, which may enhance their functional value in promoting gastrointestinal health and supporting dietary fiber intake [26]. In a study conducted on 16 types of rowanberry pomace, Sarv et al. [27] reported a mean fiber value of 15.64%, much higher than the values found in the present study in rowanberry fruits (9.51%). The findings of our study showed an elevated content of protein and ash in hawthorn fruits compared to those reported by Guo et al. [28] in a research study regarding the proximate and phytochemical composition of two cultivars of hawthorn fruit. These differences may be caused by genetic factors, state of ripening, soil structure, climatic factors and other environmental factors [29].

Ash content did not differ significantly between the two fruits. However, pronounced variations were observed in the concentrations of the individual trace elements. Hawthorn fruits contained significantly higher levels of copper, iron, and zinc, suggesting a superior contribution to micronutrient intake. The essential role of these trace elements is well known, as they act as cofactors for numerous enzymes, are involved in oxygen transport throughout the body and contribute to the proper functioning of the immune system [30,31]. Conversely, rowanberry fruits exhibited a markedly higher manganese content, a mineral essential for antioxidant defense mechanisms (e.g., manganese superoxide dismutase) and metabolic regulation [32,33]. A similar content of zinc (8.61 mg/kg) was found in rowanberry fruits by Aslantas et al. [34], compared to 9 mg/kg analyzed in the present study. The same authors reported higher values for other minerals studied in the rowanberry fruits: 2.94 mg/kg vs. 0.06 mg/kg copper, 24.20 mg/kg vs. 4.85 mg/kg iron, except manganese which was much lower than the content observed in our study (5.03 mg/kg vs. 70.02 mg/kg). In a study conducted by Liu et al. [35], it was reported that altitude has a significant impact on hawthorn’s nutritional, bioactive, and mineral profiles. Low-altitude plants, such as those analyzed in the present study, had significantly higher levels of bioactive compounds, suggesting that their biosynthesis is facilitated by moderate environmental conditions with optimal light intensity, temperature, and precipitation. On the contrary, for the high-altitude samples, an accumulation pattern of essential mineral elements was observed by the same researchers.

The substantial variability observed in the analytical results reported across scientific studies can be attributed to multiple biological and environmental factors. As previously documented, differences in the phytochemical profile of rowanberries may arise from genetic determinants, notably the pronounced tendency for apomixis within the Sorbus genus [36]. Furthermore, given the well-established role of secondary metabolites in plant adaptation and defense against biotic and abiotic stressors [37], the chemical composition of plant materials is strongly influenced by geographical origin and associated environmental conditions [38]. Variations in climate, soil characteristics, altitude, and exposure to stress factors can significantly change the morphology, physiology, and biochemistry of plants [39,40]. Consequently, such environmental and genetic heterogeneity may explain the wide range of analytical values reported for rowanberry and hawthorn fruits in the scientific literature.

Regarding the fatty acid composition of the analyzed fruits, data have shown that the PUFA/SFA ratio was significantly higher in rowanberry fruits (3.69) than in hawthorn fruits (2.91), indicating a more favorable lipid profile from a cardiovascular health perspective [41]. However, the more balanced n-6/n-3 ratio observed in hawthorn fruits suggests a potentially superior nutritional quality regarding inflammatory modulation [42]. A similar content of linoleic acid (51.94%), oleic acid (20.55%) and palmitic acid (12.96%) was observed in a study on rowanberry pomace [27]. To the best of our knowledge, data regarding the fatty acid profile of hawthorn fruits is currently limited. In the present study, hawthorn fruits exhibited a fatty acid profile dominated by linoleic acid, oleic acid and palmitic acid. A similar fatty acid pattern has been reported in other berries, where linoleic and oleic acids were the major fatty acids [43]. This type of profile may be associated with positive nutritional attributes, due to the fact that polyunsaturated and monounsaturated fatty acids play crucial roles in membrane integrity and metabolic regulation [44,45].

Lutein, zeaxanthin, astaxanthin and canthaxanthin were identified in the saponified extract of rowanberry and hawthorn fruits following analysis by RP-HPLC. In contrast to the present study, other authors [46] have reported a wider range of carotenoids in rowanberries, including all-trans-β-carotene, β-cryptoxanthin, and γ-carotene, in addition to zeaxanthin, which was also detected in the present study. In a study conducted by Bobinaitė et al. [47], the total carotenoid content recovered from dried rowanberry pomace by Soxhlet extraction was 78.91 ± 3.50 mg/100 g dw, while the β-carotene content was 38.69 ± 1.43 mg/100 g dw. O’Sullivan et al. [48] found that rowanberries contained β-cryptoxanthin at a concentration of 35 mg/100 g. The same authors have shown that wild berries, including rowanberries, are a good source of certain carotenoids that are moderately bioaccessible and therefore available for absorption by intestinal cells. The carotenoid analysis of three *Crataegus* species from Türkiye showed a significant biochemical diversity, with lutein content ranging from 0.48 to 1.37 mg/g dw, β-cryptoxanthin content from 0.47 to 0.56 mg/g dw, and β-carotene from 1.36 to 2.28 mg/g dw [49]. A screening of the carotenoids in hawthorn fruits from Romania [50] revealed the presence of mutatoxanthin, lutein, α-cryptoxanthin, β-cryptoxanthin, cis-β-carotene, all-trans-β-carotene, and lycopene, with lutein values lower than those obtained in the present study (0.64 mg/kg vs. 21.69 mg/kg). Due to their antioxidant and anti-inflammatory properties, carotenoids can protect against cognitive decline and neurodegenerative diseases, such as Alzheimer’s disease and Parkinson’s disease [51,52]. Epidemiological studies have shown that dietary lutein and zeaxanthin have a positive effect in reducing the risk of age-related macular degeneration (AMD) [53]. Moreover, it was reported that a higher consumption of carotenoids can substantially lower the risks of stroke and CVDs [54]. The results of the present study contribute to the limited data available for these fruit species and highlight their potential value as sources of health-promoting bioactive compounds.

Same as carotenoids, tocopherols are lipophilic antioxidants, which can be found in berries, contributing to the protection of cell membranes against oxidative damage and enhancing the antioxidant capacity of the fruits [55]. The results of this study showed that α-tocopherol was the most abundant isomer in both rowanberry and hawthorn fruits, with higher levels observed in hawthorn. Rowanberries exhibited an elevated content of γ-tocopherol, whereas no significant differences were noted in the concentration of total vitamin E between the two fruit types. There is a wide range of values reported in the literature for the α-tocopherol content in rowanberries. Šavikin et al. [56] found 0.48–19.85 mg/kg dw α-tocopherol in rowanberry fruits, whereas Orsavová et al. [57] reported 1.42–4.77 mg/kg vitamin E in several Sorbus cultivars. However, both studies reported lower values compared to those obtained in the present study. Similar vitamin E values were reported by Oancea et al. [58] in a study conducted on the whole hawthorn plant. The same authors showed that the ratio between tocopherol isomers is important when assessing antioxidant potential, reporting a value of 0.25 for the whole hawthorn plant. In the present study, the ratio calculated as (γ + δ)/α, was 0.38 for hawthorn fruits, while a value of 0.92 was obtained for rowanberries. According to Athanasiadis et al. [59], values close or below 1 indicate higher antioxidant activity, suggesting that a higher α-tocopherol content is associated with increased antioxidant activity. Furthermore, Zaunschirm et al. [60] demonstrated that besides the quantity of tocopherol isomers, their ratio is a valuable parameter for optimizing the oxidative stability of foods, ensuring not only the sensory properties, but also the nutritional quality of the products. In the present study, rowanberries exhibited a higher tocopherol ratio compared to hawthorn fruits, despite comparable total vitamin E contents. This variation was further reflected in the antioxidant activity results.

### 4.2. Polyphenol Profile and In Vitro Bioaccessibility Assessment

The comparative analysis of the polyphenol composition of selected wild fruits revealed important differences in polyphenol content. The polyphenol profile of hawthorn fruits showed that flavonoids were the most abundant class of compounds, which was consistent with previous reports on hawthorn fruits and other *Crataegus* species [4]. Due to the presence of quercetin, catechin, rutin and hyperoside, which are considered α-glucosidase inhibitors, hawthorn berries have been shown to exert hypoglycemic effect [61]. Additionally, a wide range of phenolic acids has been identified in hawthorn fruits. Similar results were reported by Cristea et al. [50] in a study on hawthorn berries with comparable levels of ferulic acid (1.1 mg/100 g), gallic acid (0.9 mg/100 g), and catechin (4.2 mg/100 g) to those observed in the present study.

It has been shown that cyanidin glucosides are commonly found in rowanberries [62]. In the present study, a concentration of 0.468 mg/g dw was found, which was less than 1 mg/g dw as reported by Hukkanen et al. [63]. Furthermore, in rowanberry pomace, cyanidin-3-glucoside has been identified as the major anthocyanin, accounting for up to 97% of total anthocyanins [64]. In rowanberries, flavonols were predominantly represented by rutin, which was 47 mg/kg. This was consistent with other studies, whereas rutin content ranged from 9.8 mg/kg to 71.1 mg/kg in different rowanberry cultivars [57]. The quercetin level observed in rowanberries (10 mg/kg) was higher than the one reported by Orsavová et al. [57] (2.4 mg/kg), but consistent with the 2.8–83.5 mg/kg range previously reported for 26 rowanberry cultivars originating from Serbia and Montenegro [56]. Besides quercetin, ellagic acid was found in both fruit types, which can be considered a valuable trait, due to the fact that both polyphenols have anticancer properties, and their activity is synergistic. In a study conducted by Mertens-Talcott et al. [65] it has been demonstrated that ellagic acid and quercetin together have a greater effect on human leukemia cells, exceeding that expected from a simple additive effect.

Studies on the benefits of polyphenols in nutrition and nutraceuticals rely on the research on digestion and intestinal absorption, since the most widespread polyphenols in foods and beverages are not necessarily the most bioaccessible [66].

The release of polyphenols from rowanberry and hawthorn fruits was monitored during in vitro digestion, and it was compared with the polyphenol content in the nondigested fruits, to subsequently estimate their bioaccessibility indices. In this study, each type of fruit was recovered at different stages of gastrointestinal digestion: the oral phase (OP), gastric phase (GP), and intestinal phase (IP). Some studies in the literature omit the oral phase entirely and start the digestion simulation at the gastric phase. Furthermore, there are different approaches regarding the inclusion, or not, of amylase during the oral phase [67]. Differences in how amylase is applied in the oral phase of the INFOGEST protocol highlight the variety of methodological strategies used to simulate human gastrointestinal digestion [68]. Excluding the oral phase may be done for simplicity or to focus specifically on gastric processes. In the current study, a simulated oral phase was applied, as this step is considered essential for exposing the fruit components to salivary fluids and to α-amylase activity. Qu et al. [69] reported that oral phase plays an important role in the solubilization of polyphenols from the food matrix. Exposure to salivary conditions and the action of salivary enzymes facilitates the liberation of phenolic compounds from the matrix. In particular, α-amylase activity has been shown to play a relevant role in modifying the polysaccharide network of foods, enhancing the accessibility of polyphenols to subsequent digestive processes.

Hawthorn fruits exhibited generally higher intestinal bioaccessibility values for polyphenolic compounds compared to rowanberries. Low values of polyphenol bioaccessibility were observed for both fruits in the oral phase, showing a limited release in the oral step. The percentages of polyphenolic bioaccessibility in this study are comparable to those found in blueberries by Muñoz-Fariña et al. [70]. The authors reported a 31.1% bioaccessibility of polyphenols in the oral phase for the freeze-dried samples, and 34.9% for convective-dried samples. Limited research has been published on the bioaccessibility of polyphenols in hawthorn fruits, and to the best of our knowledge, this is the first study to investigate the bioaccessibility of phenolic compounds in rowanberry fruits. Moreover, several studies have reported that berry phenolics undergo changes in bioaccessibility during digestion, providing a useful framework for understanding the digestive behavior of polyphenols in similar fruit matrices [10,20,71].

Studies have shown that the release of phenolics during gastric digestion of hawthorn fruits is higher than that of intestinal digestion [72,73], which is consistent with the results of the present study. The results showed that, during the gastric phase, the bioaccessibility of flavonoids such as epigallocatechin, catechin, epicatechin, rutin, quercetin, and cyanidin-3-glucoside was higher than the values observed in the intestinal phase. This difference can be possibly caused by pH changes. Phenolic compounds are relatively stable in the acidic environment of the gastric phase but are degraded in the neutral pH of the intestinal phase, in the presence of pancreatin and bile salts, leading to autooxidation reactions [74]. In this study, the intestinal bioaccessibility of both rowanberry and hawthorn fruits registered high values for hydroxybenzoic acids, in some cases exceeding 100%, which may be explained by the conversion of flavonols, catechins and proanthocyanidins into a series of small molecules, such as phenolic acids, as reported by Morzel et al. [75]. For instance, gallic acid is one of the most bioaccessible phenolic acids in berries. Several studies have explained this high release from the fruit matrix by the hydrolytic degradation of tannins or by singular hydration coupled with the carboxylation of ferulic acid [20,76]. Another possible explanation for the high level of bioaccessibility is related to the release of polyphenol molecules from proteins and other biomolecules [10].

In rowanberries, quercetin was found in the undigested fruits and was detected only in the intestinal phase. A possible explanation can be the enzymatic conversion of quercetin glycosides under intestinal conditions and the increased release from the fruit matrix. According to Lin et al. [77], the release and the bioaccessibility of quercetin is influenced by the food matrix and digestion conditions. Rowanberries exhibited a significantly higher intestinal bioaccessibility of hydroxycinnamic acids, whereas flavanols, flavonols and anthocyanins had a significantly higher intestinal bioaccessibility in hawthorn fruits. In blackberry fruits, cyanidin-3-glucoside and 2,4,6-trihydroxybenzoic acid were the two most widely released compounds during digestion [78], and a similar trend was observed in the present study, where cyanidin-3-glucoside exhibited the second-highest intestinal bioaccessibility in hawthorn fruits. Cyanidin-3-glucoside is known as a bioaccessible anthocyanins present in berries. Unlike other anthocyanins, its chemical structure favors its stability, bioaccessibility and bioavailability in the gastrointestinal tract. This fact is attributed to its glycosylated structure, which increases hydrophilicity and solubility in the aqueous digestive environment, facilitating its release into the bioaccessible fraction [79]. In the oral phase, cyanidin-3-glucoside exhibited a very low release from the hawthorn fruit matrix, which is consistent with previously published data. Moreover, it has been showed that α-glucosidase inhibitory activity is the strongest in the gastric digestion of blackberry fruits, due to the phenolic compounds, especially cyanidin-3-glucoside, which may exert beneficial effects for diabetes by helping to control blood glucose levels and support pancreatic function [78].

The bioaccessibility of polyphenols depends on several factors, such as the structure of the compound, the food matrix, interactions with other food components, and the presence of suppressors or cofactors [80]. During digestion, plant cell walls are broken down and dietary fibers can interact with polyphenols, modulating their bioaccessibility. Moreover, previous research has demonstrated that polyphenols can interact with carbohydrates, lipids, or proteins [81]. However, there are few studies on the bioavailability of phenolic compounds from fruit extracts as influenced by other components, and mixed results have been reported. In the present study, important differences in the bioaccessibility of phenolic compounds of rowanberry and hawthorn fruits were observed, which are likely due to the influence of the food matrix on their bioaccessibility during the digestion process, as also suggested by Ferreyra et al. [82].

### 4.3. Antioxidant Capacity and Inhibition of Lipid Peroxidation

To assess the effect of the identified and quantified antioxidants in the two fruits types, the antioxidant capacity of the fruits was studied using four complementary methods: ABTS, DPPH, TAC, and ICA. The results of this study revealed significantly higher values for DPPH and ABTS in hawthorn fruits, whereas rowanberries had a significantly higher total antioxidant capacity. Regarding iron chelating ability, no significant differences were observed between the two analyzed fruits. The differences found between the ABTS, DPPH, and TAC results can be explained by the distinct reaction mechanisms underlying these assays [83]. DPPH and ABTS measure the radical scavenging capacity but differ in radical type and solubility. In contrast, the TAC assay shows the overall reducing capacity of the extracts, including compounds that may not act as efficient radical scavengers. Therefore, the differences among the results reflect differences in the chemical nature, redox behavior, and relative contribution of individual antioxidant compounds present in the fruit matrices.

Higher values were found in the samples of 17 cultivars of sweet rowanberry originating from different countries (Russia, Slovakia, China, Germany) with DPPH values ranging from 13.3 mM Trolox in ‘Discolor’ to 64.6 mM Trolox in ‘Businka’ [57]. Romanian *Sorbus aucuparia* L. fruits were also studied for their antioxidant potential. The findings pointed out the increased antioxidant capacity of the Romanian fruits compared to several Serbian and Polish cultivars, highlighting also the antibacterial activity towards several microbial strains [2]. In this study, hawthorn fruits exhibited a DPPH value of 11.49 mM Trolox, higher than the results (3.50 mM Trolox) reported by Tamayo-Vives et al. [84] and more close to the values (13.72 mM Trolox) observed by Moldovan et al. [85].

In order to assess the antioxidant effect of the bioactive compounds found in the analyzed fruits, lipid peroxidation was induced using eggs as a biological model. Egg yolk homogenates were oxidated using the iron-ascorbic acid system, followed by oxidative inhibition with synthetic antioxidants (EDTA, BHT, vitamin E) and extracts of rowanberry and hawthorn fruits, and quantified by assaying TBARS values. The peroxidized homogenate (PH) had a higher oxidation rate than the reference values homogenate (H) recorded before incubation, showing the successful induction of lipid peroxidation. The addition of synthetic antioxidants, such as EDTA, BHT, and vitamin E to the peroxidized homogenates led to significantly lower MDA values, with no differences in the effect of the synthetic antioxidants. The supplementation of peroxidized homogenates with extracts of rowanberries (PHR) and hawthorn fruits (PHH) showed reduced MDA values compared to the peroxidized homogenates, although the antioxidant effect of the extracts was lower than that observed for the synthetic antioxidants. The differences in effectiveness between fruit extracts and synthetic antioxidants can be partly attributed to differences in purity, concentration, and chemical specificity of the active compounds. Synthetic antioxidants are purified substances with targeted mechanisms of action, whereas fruit extracts represent complex mixtures in which active compounds are present at variable concentrations and may interact synergistically or antagonistically. This complexity may contribute to their comparatively lower but still measurable antioxidant efficacy.

Hawthorn fruits exhibited a higher inhibitory effect against lipid peroxidation than rowanberry fruits. The elevated antioxidant efficiency of hawthorn fruits can be attributed to their significantly higher antioxidant capacity, as measured by ABTS and DPPH assays. Qiao et al. [86] have demonstrated that the hawthorn extract can delay the browning process of fresh-cut potatoes during storage. In agreement with our results, the same authors noted an increase in the DPPH radical scavenging activity of the hawthorn extract, and also an enhanced CAT activity, with effects in regulating the lipoxygenase (LOX) activity and in the accumulation of malondialdehyde (MDA). Shu et al. [87] highlighted the important role of hawthorn extract rich in polyphenols in improving the body status and also the effect in extending the shelf life of foods.

The findings of this study indicate that both fruit extracts succeed in inhibiting lipid peroxidation in egg yolks. Similar results were reported by Kylli and Nohynek [88], who showed that the phenolics derived from wild rowanberries were significantly effective at inhibiting lipid oxidation in liposomes and in emulsions. Moreover, Rutkowska et al. [89] demonstrated that rowanberry extracts were able to significantly protect plasma lipids against peroxidation induced by peroxynitrite, the effects being dose-dependent and moderately correlated with their phenolic contents.

This study has several limitations. First, the assessment of polyphenol bioaccessibility was performed using an in vitro simulated gastrointestinal digestion model, which cannot fully replicate the complexity of human digestion, including absorption, metabolism, and interactions with gut microbiota. Consequently, the results do not allow direct conclusions regarding the physiological effects. The egg yolk model provides a useful experimental system to study lipid peroxidation and the antioxidant effects of the selected fruits, but it has several limitations. It does not fully replicate the dynamic conditions present in living systems. Moreover, the egg yolk system has limited predictive power and cannot fully capture the interactions of lipids with proteins, metals, or other biomolecules found in vivo. Finally, several compounds on the polyphenol chromatogram could not be identified due to the absence of mass spectral data, being a limitation of the present study.

This study provides insight into the bioaccessibility of phenolic compounds in the rowanberry and hawthorn fruits, which is relevant for understanding how these compounds behave during digestion. Understanding these mechanisms helps in predicting the potential health benefits of the fruits, and can contribute to the development of functional foods, dietary supplements, or fruit-based formulations. In this study, the fruits were analyzed as whole matrices, without seed separation, in order to reflect a realistic processing scenario commonly used for dried fruit powders and extracts. Moreover, these results may have practical implications for animal nutrition. Incorporating polyphenol-rich fruits, such as hawthorn or rowanberry, into animal feed as natural additives could offer several benefits, including enhancement of the nutritional quality of animal products, modulation of gut health, reduction in oxidative stress, and improvement of overall digestive efficiency in livestock. The consideration of animal nutrition is presented as a potential valorization pathway for these underutilized fruits and their derived products, without implying direct extrapolation to animal digestive physiology.

While the results indicate a higher bioaccessibility and antioxidant potential for hawthorn compared to rowanberry, confirmation through in vivo studies is necessary before making definitive claims regarding health benefits. Moreover, practical doses, processing stability, and sensory acceptability should be addressed in future in vivo studies.

## 5. Conclusions

Rowanberry and hawthorn fruits were demonstrated to be rich in bioactive compounds, such as xanthophylls, vitamin E and phenolic compounds. The benefits of polyphenols in nutrition are strongly related to the bioaccessibility and fate of polyphenol compounds during digestion. Hawthorn fruits generally exhibited higher intestinal bioaccessibility values for polyphenolic compounds compared to rowanberries. Cyanidin-3-glucoside was the second most abundant intestinally bioaccessible polyphenol in hawthorn fruits. Both fruits contained quercetin and ellagic acid, the latter having an elevated bioaccessibility, which is considered a valuable trait, as both polyphenols have anticancer properties and their activity is synergistic.

Rowanberry and hawthorn fruits exhibited an inhibitory effect on lipid peroxidation in yolk homogenates, although their efficiency was below that of the synthetic antioxidants used as controls in this study. The elevated antioxidant efficiency of hawthorn fruits compared to rowanberries can be attributed to their significantly higher antioxidant capacity, as measured by ABTS and DPPH assays. The findings of this study show that rowanberry and hawthorn fruits can be considered promising sources of bioactive compounds like bioaccessible polyphenols, with demonstrated antioxidant efficiency in biological models. These results can serve for the potential valorization of both fruits in the development of nutraceuticals, in animal nutrition for the development of functional foods of animal origin, and in any health-oriented food systems.

## Figures and Tables

**Table 1:** Proximate composition and mineral content in rowanberry and hawthorn fruits.

Specification	Rowanberry Fruits	Hawthorn Fruits	SEM	*p*-Value
Dry matter (%)	87.64 b	89.35 a	0.446	0.030
Crude protein (%)	5.28 a	4.12 b	0.283	0.011
Crude fat (%)	2.96 a	2.22 b	0.184	0.014
Carbohydrates (%)	66.35 a	49.14 b	3.880	<0.0001
Crude fiber (%)	9.51 b	30.53 a	4.707	<0.0001
Ash (%)	3.55 a	3.34 a	0.093	0.307
*Minerals*				
Copper (mg/kg dw)	0.00 * b	2.89 a	0.633	<0.0001
Iron (mg/kg dry dw)	4.85 b	69.59 a	14.479	<0.0001
Manganese (mg/kg dry dw)	70.02 a	7.28 b	14.032	<0.0001
Zinc (mg/kg dry dw)	9.00 b	11.51 a	0.578	<0.0001

* Not detectable. Statistical analysis was performed using one-way ANOVA followed by Tukey’s post hoc test (*p* < 0.05). Different letters within the same row indicate significant differences.

**Table 2:** Fatty acid profile of rowanberry and hawthorn fruits (g/100 g Total FAs).

Fatty Acids	C:D	Rowanberry Fruits	Hawthorn Fruits	SEM	*p*-Value
Capric acid	C 10:0	0.123 a	0.115 a	0.002	0.081
Lauric acid	C 12:0	0.098 b	0.110 a	0.002	0.001
Myristic acid	C14:0	0.285 a	0.255 a	0.008	0.216
Pentadecanoic acid	C15:0	0.115 a	0.125 a	0.002	0.070
Pentadecenoic acid	C15:1	0.260 a	0.255 a	0.007	0.824
Palmitic acid	C16:0	10.46 b	11.79 a	0.211	0.0001
Palmitoleic acid	C16:1	0.360 b	0.540 a	0.030	0.005
Heptadecanoic acid	C17:0	0.105 a	0.110 a	0.001	0.158
Heptadecenoic acid	C17:1	0.145 b	0.205 a	0.011	0.023
Stearic acid	C18:0	2.795 b	3.275 a	0.077	0.000
Oleic acid	C18:1n-9	25.60 a	25.48 a	0.051	0.492
Linoleic acid	C18:2n-6	56.10 a	50.74 b	0.852	<0.0001
α Linolenic acid	C18:3n-3	0.860 b	1.350 a	0.078	<0.0001
γ Linolenic acid	C18:3n-6	0.065 a	0.090 b	0.004	<0.0001
Octadecatetraenoic acid	C18:4n-3	0.425 b	0.955 a	0.084	<0.0001
Arachic acid	C20:0	0.019 a	0.000 b	0.003	<0.0001
Eicosadienoic acid	C20:2n-6	0.055 b	0.460 a	0.064	<0.0001
Eicosapentaenoic acid	C20:5n-3	0.000 b	0.365 a	0.058	<0.0001
Arachidonic acid	C20:4n-6	0.135 a	0.000 b	0.022	<0.0001
Tricosanoic acid	C23:0	1.565 b	2.365 a	0.127	<0.0001
Docosadienoic acid	C22:2n-6	0.000 b	0.170 a	0.027	<0.0001
Docosatrienoic acid	C22:3n-6	0.000 b	0.085 a	0.013	<0.0001
Docosatetraenoic acid	C22:4n-6	0.645 a	0.000 a	0.137	0.091
Lignoceric acid	C24:0	0.000 b	0.515 a	0.081	<0.0001
Σ SFA		15.57 b	18.66 a	0.692	<0.0001
Σ MUFA		26.36 a	26.30 a	0.096	0.792
Σ PUFA		57.44 a	54.22 b	0.732	0.0004
Σ n-3		1.28 b	2.67 a	0.310	<0.0001
Σ n-6		57.00 a	51.55 b	1.245	0.001
n-6/n-3		44.39 a	19.31 b	5.633	<0.0001
PUFA/SFA		3.69 a	2.91 b	0.176	<0.0001

FAs, fatty acids; C:D, carbon number: double bounds number; SFA, saturated fatty acids; UFA, total unsaturated fatty acids; MUFA, monounsaturated fatty acids; PUFA, polyunsaturated fatty acids. The relative concentration of each fatty acid is reported as gram of fatty acids/100 g of total fatty acids. Statistical analysis was performed using one-way ANOVA followed by Tukey’s post hoc test (*p* < 0.05). Different letters within the same row indicate significant differences.

**Table 3:** The content of liposoluble antioxidants (μg/g) analyzed in rowanberry and hawthorn fruits.

Specification	Rowanberry Fruits	Hawthorn Fruits	SEM	*p*-Value
*Tocopherols*				
α-tocopherol	34.05 b	47.30 a	2.978	<0.0001
δ-tocopherol	11.48 a	11.10 a	0.169	0.314
γ-tocopherol	19.74 a	6.813 b	2.894	<0.0001
Total vitamin E	65.27 a	65.55 a	0.387	0.759
*Xantophylls*				
Lutein	20.19 b	21.69 a	0.415	0.050
Astaxanthin	1.527 a	0.897 a	0.205	0.131
Canthaxanthin	1.722 a	1.072 b	0.148	0.001

Statistical analysis was performed using one-way ANOVA followed by Tukey’s post hoc test (*p* < 0.05). Different letters within the same row indicate significant differences.

**Table 4:** Polyphenol profile (mg/g) of rowanberry fruits after simulated in vitro gastrointestinal digestion.

Specification	Rowanberry Fruits						
	BD	OP	BI (%)	GP	BI (%)	IP	BI (%)
**Phenolic acids**							
*Hydroxybenzoic acids*							
Gallic acid	0.034	0.017	49.98	0.028	83.12	0.022	66.03
Vanillic acid	0.029	0.016	55.09	0.015	53.30	0.030	103.65
Syringic acid	0.022	0.014	62.90	0.019	86.56	0.022	99.72
3-Hydroxybenzoic acid	0.019	0.008	43.83	0.010	50.56	0.017	92.28
Ellagic acid	0.009	0.004	46.23	0.008	96.61	0.008	95.47
Protocatechuic acid	0.001	0.001	46.76	0.001	58.49	0.001	67.69
*Hydroxycinnamic acids*							
Chlorogenic acid	0.003	0.002	51.49	0.002	75.62	0.003	81.42
Caffeic acid	0.022	0.005	20.74	0.006	26.98	0.012	54.59
p-Methoxycinnamic acid	0.033	0.0005	1.38	0.003	8.02	0.020	60.93
Ferulic acid	0.024	0.011	44.52	0.015	60.47	0.020	85.14
Coumaric acid	0.003	0.002	50.92	0.003	89.74	0.003	91.75
Trans-cinnamic acid	0.004	0.002	48.33	0.002	62.26	0.005	119.21
**Flavonoids**							
*Flavanols*							
Epigallocatechin	0.451	0.048	10.55	0.164	36.29	0.026	5.75
Catechin	4.361	2.360	54.10	3.422	78.47	3.797	87.05
Epicatechin	0.058	0.029	50.24	0.049	83.64	0.033	57.38
*Flavonols*							
Rutin	0.047	0.016	34.18	0.015	31.47	0.008	17.78
Quercetin	0.010	nd	0.00	nd	0.00	0.002	23.19
*Anthocyanins*							
Cyanidine-3-glucoside	0.468	0.185	39.44	0.245	52.28	0.311	66.44
**Stilbenes**							
Resveratrol	0.001	0.0002	30.22	0.0003	39.06	0.001	93.21

BD = before digestion, OP = oral phase, GP = gastric phase, IP = intestinal phase, BI = bioaccessibility index, nd = not detected.

**Table 5:** Polyphenol profile (mg/g) of hawthorn fruits after simulated in vitro gastrointestinal digestion.

Specification	Hawthorn Fruits						
	BD	OP	BI (%)	GP	BI (%)	IP	BI (%)
**Phenolic acids**							
*Hydroxybenzoic acids*							
Gallic acid	0.017	0.006	34.64	0.018	103.10	0.028	164.48
Vanillic acid	0.013	0.005	35.76	0.008	60.65	0.011	84.29
Syringic acid	0.056	0.013	23.43	0.048	85.19	0.065	115.22
3-Hydroxybenzoic acid	0.014	0.003	24.16	0.007	50.75	0.006	44.10
Ellagic acid	0.010	0.004	43.64	0.008	78.83	0.011	108.93
Protocatechuic acid	0.002	0.0005	18.78	0.0002	7.22	0.001	39.27
*Hydroxycinnamic acids*							
Chlorogenic acid	0.006	0.002	33.56	0.004	73.86	0.004	66.98
Caffeic acid	0.005	0.002	31.99	0.004	70.14	0.004	84.52
p-Methoxycinnamic acid	0.001	0.0002	24.97	0.001	77.97	0.001	96.28
Ferulic acid	0.013	0.001	8.05	0.011	78.26	0.012	90.35
Coumaric acid	0.021	0.004	19.49	0.006	27.76	0.018	86.17
Trans-cinnamic acid	nd	nd	0.00	nd	0.00	nd	0.00
**Flavonoids**							
*Flavanols*							
Epigallocatechin	0.263	0.065	24.76	0.131	49.97	0.064	24.43
Catechin	0.220	0.067	30.21	0.224	101.79	0.156	70.61
Epicatechin	0.043	0.012	28.03	0.042	98.71	0.041	96.86
*Flavonols*							
Rutin	0.020	0.006	29.35	0.013	65.69	0.016	81.09
Quercetin	0.003	0.0001	2.02	0.003	80.56	0.001	35.57
*Anthocyanins*							
Cyanidine-3-glucoside	0.081	0.002	2.76	0.111	137.06	0.106	130.46
**Stilbenes**							
Resveratrol	0.0004	0.0001	35.71	0.0003	96.90	0.0003	96.82

BD = before digestion, OP = oral phase, GP = gastric phase, IP = intestinal phase, BI = bioaccessibility index, nd = not detected.

**Table 6:** Effect of rowanberry and hawthorn fruit extracts on phospholipids peroxidation in eggs.

Specification	Antioxidant Concentration	MDA, mg/kg
Homogenate (H)	-	4.58 e
Peroxidized homogenate (PH)	-	50.79 a
Peroxidized homogenate + EDTA (PHEDTA)	1000 mg/kg	9.12 d
Peroxidized homogenate + BHT (PHBHT)	1000 mg/kg	11.05 d
Peroxidized homogenate + vit E (PHVE)	1000 mg/kg	12.12 d
Peroxidized homogenate + hawthorn (PHH)	1000 mg/kg *	20.58 c
Peroxidized homogenate + rowanberry (PHR)	1000 mg/kg *	37.19 b
SEM		3.116
*p*-value		<0.0001

* Concentration of the extracts. Statistical analysis was performed using one-way ANOVA followed by Tukey’s post hoc test (*p* < 0.05). Different letters within the column indicate significant differences.

## Data Availability

The original contributions presented in this study are included in the article/Supplementary Material. Further inquiries can be directed to the corresponding author.

## Supplementary Materials

The following supporting information can be downloaded at: https://www.mdpi.com/article/10.3390/antiox15030349/s1, Figure S1. Chromatogram of the carotenoid standards (overlay) 1-astaxanthin, 2-lutein, 3-canthaxanthin. Description of extract preparation for polyphenol analysis. Description of extract preparation for antioxidant activity analysis. Description of the simulated digestion phases.
