# Supplementary material for: Bromatological Profile of Fruits from Sorbus aucuparia and Crataegus monogyna: Polyphenol Bioaccessibility and Inhibitory Effect on Lipid Peroxidation in a Biological Model
Source: Antioxidants (Basel). 2026 Mar 11;15(3):349. doi: 10.3390/antiox15030349 (PMC13024522; doi:10.3390/antiox15030349)
Supplement: Supplementary file 1 [file antioxidants-15-00349-s001.zip › antioxidants-4140379-supplementary.pdf]

Preparation of the extracts for polyphenol analysis

The preparation of the extracts for polyphenol analysis was performed with a mixture of water/methanol/acetic acid in a ratio of 69:30:1 (v/v/v). The samples were incubated for 60 min in a shaking water bath (Memmert, Schwabach, Germany) at 50 °C. Afterwards, samples were centrifuged at 4000 rpm for 15 min, and the supernatant was collected.

Preparation of the extracts for antioxidant activity analysis

For the determination of the antioxidant capacity, the plant materials were macerated with 80% methanol for 24 h at room temperature in the dark. Then, the samples were centrifuged (1500 g for 10 min) and the supernatant was collected for further analysis.

Preparation of the digestion phases

The simulated salivary fluid (SSF) contained 15.1 mM KCl, 3.7 mM KH_2PO_4, 13.6 mM $NaHCO_3$, 0.15 mM $MgCl_2$ $(H_2O)_6$, 0.06 mM $(NH_4)_2CO_3$, and 1.5 mM $CaCl_2$. The simulated gastric fluid (SGF) was formulated with 6.9 mM KCl, 0.9 mM KH_2PO_4, 25 mM $NaHCO_3$, 47.2 mM NaCl, 0.10 mM $MgCl_2$ $(H_2O)_6$, 0.50 mM $(NH_4)_2CO_3$, and 0.15 mM $CaCl_2$. The simulated intestinal fluid (SIF) consisted of 6.8 mM KCl, 0.8 mM KH_2PO_4, 85 mM $NaHCO_3$, 38.4 mM NaCl, 0.33 mM $MgCl_2$ $(H_2O)_6$, and 0.6 mM $CaCl_2$.

The following enzymes were used: α-amylase (prepared in SSF, final concentration 75 U/mL), pepsin solution (prepared in SGF, final concentration 2000 U/mL), pancreatin at 800 U/mL (prepared in SIF, final concentration 100 U/mL), bile salts at 160 mM (final concentration 10 mM).

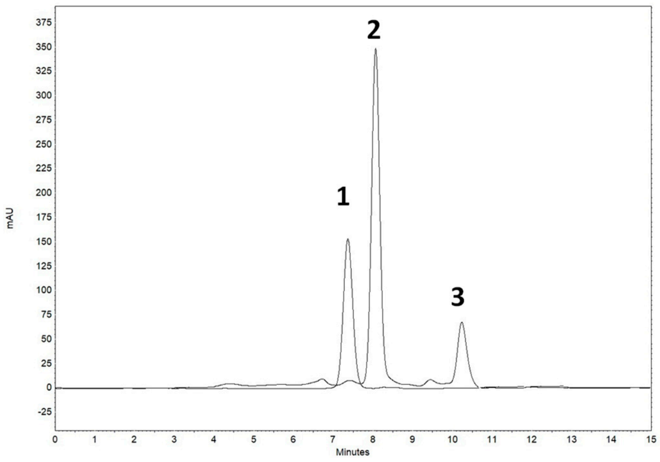

Figure S1. Chromatogram of the carotenoid standards (overlay) 1-astaxanthin, 2-lutein, 3-canthaxanthin.
